# Fast Brain Plasticity during Word Learning in Musically-Trained Children

**DOI:** 10.3389/fnhum.2017.00233

**Published:** 2017-05-12

**Authors:** Eva Dittinger, Julie Chobert, Johannes C. Ziegler, Mireille Besson

**Affiliations:** ^1^Laboratoire de Neurosciences Cognitives (LNC, UMR 7291), CNRS, Aix-Marseille UniversityMarseille, France; ^2^Laboratoire Parole et Langage (LPL, UMR 7309), CNRS, Aix-Marseille UniversityAix-en-Provence, France; ^3^Laboratoire de Psychologie Cognitive (LPC, UMR 7290), CNRS, Aix-Marseille UniversityMarseille, France

**Keywords:** word learning, musical expertise, picture-word associations, semantic memory, N400

## Abstract

Children learn new words every day and this ability requires auditory perception, phoneme discrimination, attention, associative learning and semantic memory. Based on previous results showing that some of these functions are enhanced by music training, we investigated learning of novel words through picture-word associations in musically-trained and control children (8–12 year-old) to determine whether music training would positively influence word learning. Results showed that musically-trained children outperformed controls in a learning paradigm that included picture-sound matching and semantic associations. Moreover, the differences between unexpected and expected learned words, as reflected by the N200 and N400 effects, were larger in children with music training compared to controls after only 3 min of learning the meaning of novel words. In line with previous results in adults, these findings clearly demonstrate a correlation between music training and better word learning. It is argued that these benefits reflect both bottom-up and top-down influences. The present learning paradigm might provide a useful dynamic diagnostic tool to determine which perceptive and cognitive functions are impaired in children with learning difficulties.

## Introduction

Learning new words is a specifically human faculty that mobilizes several perceptual and cognitive abilities: sound perception and discrimination, attention, associative learning, and semantic memory (Perfetti et al., 2005; Davis and Gaskell, 2009). Here, we investigated the temporal dynamics of word learning in a novel word learning paradigm using both behavioral measures and Event-Related Potentials (ERPs). The main question was whether musically-trained children would outperform controls in terms of novel word learning and semantic association skills and whether this would be reflected in dynamic brain plasticity measures (ERPs).

It is well known that music training improves auditory perception and attention (Kraus and Chandrasekaran, 2010). Moreover, it has been shown that music training also enhances several aspects of language processing, including phoneme and syllable perception (Musacchia et al., 2007; Chobert et al., 2011, 2014; Marie et al., 2011; Elmer et al., 2012, 2014; Parbery-Clark et al., 2012; Kühnis et al., 2013; Bidelman et al., 2014), the processing of pitch and prosody (Schön et al., 2004; Thompson et al., 2004; Delogu et al., 2006; Magne et al., 2006; Marques et al., 2007; Wong et al., 2007; Lima and Castro, 2011; Bidelman et al., 2013), phonological processing and reading (Anvari et al., 2002; Moreno et al., 2009; Corrigall and Trainor, 2011; Huss et al., 2011), speech segmentation (François et al., 2013), and syntactic processing (Jentschke and Koelsch, 2009; Gordon et al., 2015). However, it remains an open question whether music training improves associative learning and semantic memory, two of the processes that are at the heart of word learning but that are not necessarily language-specific (Markson and Bloom, 1997).

The ERP methodology is well-suited to examine the temporal dynamics of word learning and brain plasticity as reflected by modulations of ERP components. Previous research on word learning has shown that the N400, a negative-going component that typically develops between 300 ms and 600 ms post-stimulus onset (Kutas and Hillyard, 1980), increased in amplitude when meaningless items acquire meaning and then decreased with further repetitions. This effect has been demonstrated in infants (from 9–24 months old; Friedrich and Friederici, 2008; Torkildsen et al., 2009; Junge et al., 2012; Borgström et al., 2015; Friedrich et al., 2015) and in adults (see below). However, to our knowledge, it has not yet been investigated in children and the present study was intended to fill this gap by testing children between 8 and 12 years old.

The increase in N400 amplitude with word learning is typically very fast. This effect has been observed in native adult English-speakers after 14 h of learning the meaning of novel French words (McLaughlin et al., 2004), after less than 1 or 2 h of learning novel word-picture associations (Dobel et al., 2009, 2010), after 45 min of learning the meaning of rare words (e.g., “clowder”; Perfetti et al., 2005) and even after a single exposure if a novel word or pseudoword was presented in a strongly constrained and meaningful context (Mestres-Missé et al., 2007; Borovsky et al., 2010, 2012; Batterink and Neville, 2011). Moreover, this fast increase in N400 amplitude is typically largest over fronto-central brain regions (FN400), as demonstrated in language-learning tasks and it possibly reflects speech segmentation and the building-up of novel word meaning (De Diego Balaguer et al., 2007; Mestres-Missé et al., 2007), two processes that may develop in parallel (François et al., 2017). While the scalp distribution of ERP components does not necessarily reflect the activation of directly underlying brain structures, it is nevertheless interesting that fronto-central brain regions are also found to be involved in the maintenance of novel information in working- or short-term memory, in the formation of new associations (Hagoort, 2014) and/or the construction of word representations in episodic memory (Wagner et al., 1998; Rodríguez-Fornells et al., 2009). Further exposures then allow for the integration of these novel items into existing lexical networks (Dumay and Gaskell, 2007), with recent results emphasizing the role of sleep in these processes (Tamminen et al., 2010, 2013; Friedrich et al., 2015). Once these “novel” representations are stabilized, the N400 is largest over centro-parietal regions as typically found for the N400 to already known words (Kutas et al., 1988).

**Figure 1:**
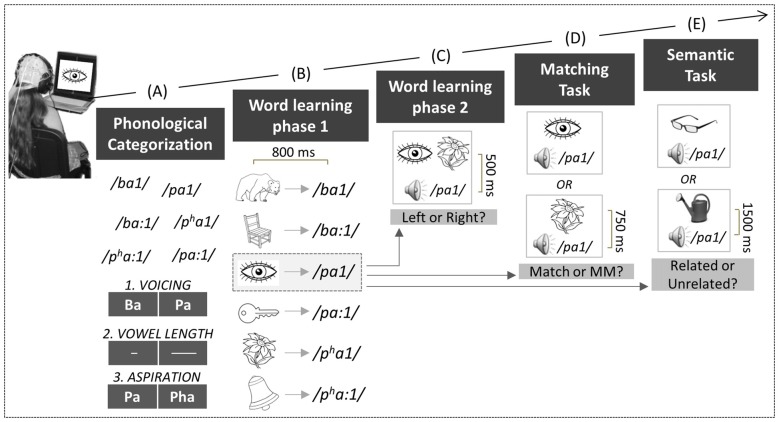
**Experimental design**. Children performed a series of tasks: First, in the phonological categorization tasks **(A)**, six natural Thai mono-syllabic words had to be categorized based on voicing (Task 1), vowel length (Task 2), or aspiration contrasts (Task 3). Second, in the word learning phase 1 **(B)**, each word was paired with its respective picture. Third, in the word learning phase 2 **(C)**, two of the pictures were presented simultaneously on the screen, together with an auditory word that matched one of the two pictures. Fourth, in the matching task **(D)**, the auditory words were presented with one of the pictures, either matching or mismatching the previously learned associations. Fifth, in the semantic task **(E)**, the auditory words were presented with novel pictures that were either semantically related or unrelated to the words.

Recently, we conducted an experiment in young adults, professional musicians and non-musicians (Dittinger et al., 2016) to test the hypothesis that music training would positively influence novel word learning. This hypothesis builds up on the results reviewed above and on recent behavioral and brain imaging results suggesting that music and language not only involve common sensory-perceptual processes (both at sub-cortical and cortical levels, Kraus and Chandrasekaran, 2010; Asaridou and McQueen, 2013) but also attentional (Patel, 2008; Tervaniemi et al., 2009; Strait et al., 2010, 2015; Perruchet and Poulin-Charronnat, 2013) and short-term memory resources (Ho et al., 2003; George and Coch, 2011) as well as executive functions (Pallesen et al., 2010; Moreno et al., 2011; Rogalsky et al., 2011; Zuk et al., 2014) that are involved in novel word learning. Results showed that adult musicians outperformed non-musicians in the most difficult task that required participants to map newly acquired words onto semantic associates. Moreover, the shift in N400 scalp distribution from frontal sites (FN400) during meaning acquisition, to parietal sites when word meaning is stabilized (N400) was faster in musicians than in non-musicians. Interestingly, results also showed that the amplitude of both the N200 and N400 components in the semantic task was larger to unrelated than to related words in musicians but not in non-musicians. The aim of the present experiment was to determine whether these results would replicate when comparing children with and without music training. The general procedure, inspired from Wong and Perrachione (2007), and the specific hypotheses are described below (see Figure 1).

Children were first asked to categorize unfamiliar Consonant-Vowel syllables with respect to voicing, vowel length and aspiration. For this purpose, we used Thai monosyllabic words. Thai is a tonal and a quantitative language in which both tonal (i.e., 5 tones) and vowel length contrasts are linguistically relevant for understanding word meaning (e.g., /pa1/ low tone with a short vowel means “to find” and /pa:1/ low tone with a long vowel means “forest”; Gandour et al., 2002). We hypothesized that if music training reinforces auditory perception and attention, children with music training should make fewer errors in the phonological categorization tasks than control children especially when the task is the most difficult, that is, for the phonological contrasts that do not belong to the French phonemic repertory (e.g., aspiration; Dobel et al., 2009).

After the phonological categorization task, the same children were then asked to learn the meaning of novel words through picture-word associations. Based on the previous results reviewed above in adults and in infants, we hypothesized that both an N200 and an N400 over frontal regions (FN400) would develop during word learning in all children but that this effect would develop faster in children with music training as was recently shown in adult musicians compared to non-musicians.

Following picture-word learning, children were then tested for the efficiency of learning using two tasks: a matching task in which they decided whether the picture-word associations matched or mismatched those seen in the word learning phase and a semantic task in which new pictures were presented that were semantically related or unrelated to the newly-learned words. Based on previous results (Meyer and Schvaneveldt, 1971), we expected mismatching and semantically unrelated words to be associated with higher error rates (ERRs) and/or slower Reaction Times (RTs) than matching and related words. Such typical semantic priming effects were predicted in all children, showing that they all learned the picture-word associations and that learning generalized to new pictures. Moreover, we expected these behavioral effects to be accompanied by larger N400s in all children to mismatching and semantically unrelated words than to matching/related words over parietal brain regions, as typically reported for the N400 to already known words in children (Holcomb et al., 1992; Juottonen et al., 1996; Hahne et al., 2004) and in adults (Kutas and Federmeier, 2011, for review). Finally, based on our previous results with adults (Dittinger et al., 2016), we predicted lower error rates and larger N200 and N400 effects (the difference between mismatching/semantically unrelated words and matching/related words) in children with music training than in controls.

## Materials and Methods

### Participants

A total of 32 children, native speakers of French without known hearing or neurological deficits participated in this experiment with 16 children that were involved in extra-scholar music training (MUS) and 16 children not involved in music training (NM), except for obligatory music lessons at school. However, participants in the NM group participated at least in one extra-scholar activity that was not related to music (e.g., sports, painting, dance), which suggests that both groups of children benefitted from stimulating extra-scholar environments.

Three children (2 with and 1 without music training) were excluded based on their level of performance in the matching and semantic tasks (i.e., percentage of error ± 2 standard deviations away from the mean) and six children (2 with and 4 without music training) because of too many artifacts in the electrophysiological data, leading to an attrition rate of 28% which is not uncommon in ERP studies with children (De Boer et al., 2005). The final group of musically-trained children (MUS) comprised six boys and six girls with three left-handers (mean age = 134.0 months, SD = 13.5) and the group of control children (NM) six boys and five girls with one left-hander (mean age = 124.5 months, SD = 20.0; *F*_(1,21)_ = 1.84, *p* = 0.19).

In the music group, children practiced music for an average of 4.9 years (4–7 years; 5 children played the piano, 2 the trumpet, 2 the trombone, 2 the violin, and 1 the saxophone). None of the children was bilingual and all children had similar socio-economic background ranging from middle to low social class as determined from the parents’ profession and according to the criteria of the National Institute of Statistics and Economic Studies (MUS: 4.4 and NM: 4.3). The protocol was approved by the local Ethical Review Committee of Aix-Marseille University, and the study was conducted in accordance with local norms and guidelines for the protection of human subjects. All children agreed to participate in the experiment once the procedure had been explained to them. Children were also told that they could stop the experiment at anytime if they felt uncomfortable (none did). Finally, at least one parent accompanied each child to the laboratory and signed an informed consent form in accordance with the Declaration of Helsinki before the experiment. Children were given presents at the end of the session to thank them for their participation. The experiment lasted for 2.5 h, which included the pose of the electrocap.

### Screening Measures

#### Cognitive and Language Abilities

Standardized psychometric tests were used to examine language-related abilities, phonological awareness (phoneme fusion), reading abilities (regular words, irregular words, pseudo-words; ODEDYS, Jacquier-Roux et al., 2005) as well as short-term memory (forward Digit Span, WISC-IV, Wechsler, 2003), visual attention (NEPSY, Korkman et al., 1998), and non-verbal intelligence (progressive matrices, PM47, Raven, 1976).

#### Musical Aptitude

Children performed two musicality tests (adapted from the MBEA battery; Peretz et al., 2003) consisting in judging whether pairs of piano melodies were same or different, based either on melodic or on rhythmic information.

### Experimental Stimuli

#### Auditory Stimuli

Six natural Thai monosyllabic words were selected for the experiment: /ba1/, /pa1/, /p^h^a1/, /ba:1/, /pa:1/, /p^h^a:1/. These words varied in vowel length, with short (/ba1/, /pa1/ and /p^h^a1/; 261 ms on average) and long vowels (/ba:1/, /pa:1/ and /p^h^a:1/; 567 ms on average), in Voice Onset Time (VOT; /ba1/ and /ba:1/, VOT = −140 ms vs. /pa1/ and /pa:1/, VOT = 5 ms), and in aspiration (/pa1/ and /pa:1/, VOT = 5 ms vs. /p^h^a1/ and /p^h^a:1/, VOT = 77 ms). Stimuli were recorded by a female Thai-French bilingual, ensuring that all words were produced naturally. For each word, five versions were digitally recorded in order to reproduce natural speech variability. Fundamental frequency was similar for each word (F0: 175 Hz) and sound pressure level was normalized across all words to a mean level of 70 dB using *Praat* software (Boersma and Weenink, 2011).

#### Visual Stimuli

For the learning phase, six pictures representing familiar objects (i.e., bear, flower, key, chair, bell, eye) were selected based on the standardized set of 260 pictures (that are matched for name and image agreement, familiarity and visual complexity) built by Snodgrass and Vanderwart (1980). The same pictures as in the learning phase were presented in the matching task. For the semantic task, 36 new pictures that the children had not seen before in the experiment and that were semantically related or unrelated to the meaning of the newly-learned words were chosen from the internet by two of the authors (JC and MB). Semantic relatedness between new and old pictures (that is, those previously presented during the word learning phase and those presented in the semantic task) was confirmed by results of pre-tests with pilot children.

### Experimental Tasks

Children were tested individually in a quiet experimental room while they sat in a comfortable chair at about 1 m from a computer screen. Auditory stimuli were presented through HiFi headphones (Sennheiser, HD590). Visual and auditory stimuli presentation, as well as the collection of behavioral data, were controlled by the “Presentation” software (NeuroBehavioral Systems, Version 11.0). Children performed six concatenated tasks (see Figure 1).

#### Phonological Categorization Task

Children performed three different phonological categorization tasks that lasted for 2.5 min each (see Figure 1A). All six Thai monosyllabic words were presented in each task using a 2500 ms Stimulus-Onset-Asynchrony (SOA). Children were asked to categorize them based upon different features in each task: (1) voicing contrast (e.g., /ba1/ vs. /pa1/); (2) vowel length (e.g., short: /ba1/ vs. long /ba:1/); and (3) aspiration contrast (e.g., /pa1/ vs. /p^h^a1/). For each task, the contrast was visually represented on the screen (see Figure 1A). Response side and task order were counterbalanced across children. Children were asked to press as quickly and as accurately as possible the left or right hand response button according to whether the auditory words matched the visual representation on the left or right side of the screen. Each monosyllabic word was presented 10 times in a pseudo-randomized order with the constraints of no immediate repetition of the same word, and no more than four successive same responses.

#### Word Learning Phase 1

Children were asked to learn the meaning of each word previously presented in the phonological categorization task through picture-word associations. No behavioral response was required, but children were asked to remember the words for subsequent tests. The picture was presented first, and then followed after 800 ms by one of the six words. For instance, a drawing of an eye was followed by the auditory presentation of the word /pa1/ and thus /pa1/ was the word for eye in our “foreign” language (see Figure 1B). Two different lists were built so that across children different pictures were associated with different words. Total trial duration was 2800 ms. Each of the six picture-word pairs was presented 20 times, resulting in 120 trials that were pseudo-randomly presented (i.e., no immediate repetition of the same association) in two blocks of 3 min each. To closely follow the brain dynamics involved in word learning, ERPs in each block were further divided into two sub-blocks for a total of four sub-blocks (i.e., Block 1: trials 1–30; Block 2: trials 31–60; Block 3: trials 61–90 and Block 4: trials 91–120).

#### Word Learning Phase 2

To consolidate learning, children performed a task in which two different pictures were simultaneously presented on the left and right sides of the screen, followed after 500 ms by one of the six words (see Figure 1C). Children were asked to press the left response key if the word matched the picture on the left side of the screen or the right key if the word matched the right-side picture (half of the stimuli in each condition). Visual feedback regarding response correctness was given, followed by the presentation of the correct picture-word pair to strengthen the association. Total trial duration was 6000 ms. Each of the six picture-word pairs was presented 10 times, resulting in 60 trials that were pseudo-randomly presented (i.e., no immediate repetition of the same association and no more than four successive same responses), within two blocks of 3 min each. Behavioral data were analyzed but not ERPs because the procedure was complex and comprised too many events.

#### Matching Task

One of the six pictures was presented, followed after a 750 ms delay by an auditory word that matched or mismatched the associations previously learned. For instance, while the drawing of an eye followed by /pa1/ was a match, the drawing of a flower followed by /pa1/ was a mismatch (see Figure 1D). Children gave their responses by pressing one out of two response keys as quickly and as accurately as possible. Some examples were given before starting the task. Response hand was counter-balanced across children. At the end of the trial, a row of X’s appeared on the screen. Children were asked to blink during this time period (1500 ms; total trial duration: 4750 ms) in order to minimize eye movement artifacts during word presentation. Each word was presented 20 times, half in the match and half in the mismatch conditions. The total of 120 trials was pseudo-randomly presented (i.e., no immediate repetition of the same association and no more than four successive same responses) within two blocks of 5 min each.

#### Semantic Task

One of the new pictures was presented, followed after 1500 ms by a semantically related or unrelated word. For instance, while the picture of glasses was semantically related to the previously learned word /pa1/ (i.e., “eye”), the picture of a watering can was semantically unrelated to /pa1/ (see Figure 1E). Children were asked to decide as quickly and as accurately as possible if the auditory word was semantically related to the new picture. Responses were given by pressing one of two response keys. Response hand was counter-balanced across participants and some examples were given before starting the task. At the end of the trial a row of X’s appeared on the screen, and children were asked to blink during this time period (1500 ms; total trial duration: 7000 ms). Each word was presented 12 times but none of the new pictures were repeated, so that on each trial the words were always associated with a different related or unrelated picture. Half of the picture-word pairs were semantically related and half were semantically unrelated. A total of 72 trials was presented pseudo-randomly (i.e., no immediate repetition of the same association and no more than four successive same responses) within two blocks of 4.2 min each.

### EEG Data Acquisition

The Electroencephalogram (EEG) was continuously recorded at a sampling rate of 512 Hz with a band-pass filter of 0–102.4 Hz by using a Biosemi amplifier system (Amsterdam, BioSemi Active 2) with 32 active Ag-Cl electrodes (Biosemi Pintype) located at standard positions according to the International 10/20 System (Jasper, 1958). EEG recordings were referenced on-line to a common electrode (CMS) included in the headcap (next to Cz). Two additional electrodes were placed on the left and right mastoids and data were re-referenced off-line to the average activity of the left and right mastoids, filtered with a bandpass filter from 0.1–40 Hz (slope of 12 dB/oct). The electro-oculogram (EOG) was recorded from flat-type active electrodes placed 1 cm to the left and right of the external canthi, and from an electrode beneath the right eye. Electrode impedance was kept below 5 kΩ. EEG data were analyzed using the Brain Vision Analyzer software (Version 1.05.0005; Brain Products, Gmbh). Independent component analysis (ICA) and inverse ICA were used to identify and remove components associated with vertical and horizontal ocular movements. Finally, baseline correction, DC-detrend and removal of artifacts above a gradient criterion of 10 μV/ms or a max-min criteria of 100 μV over the entire epoch were applied resulting in an average of 12% of rejected trials. For each child, ERPs were time-locked to word onset, and segmented (including a 200 ms baseline) into 1200 ms epochs in the phonological categorization tasks and into 1700 ms epochs in the other tasks (i.e., word learning phase 1, matching and semantic tasks). To increase the signal to noise ratio, all responses were considered to compute the individual averages.

### Statistical Analysis

Analysis of Variance (ANOVAs) were computed using the Statistica software (Version 12.0, StatSoft Inc., Tulsa). For errors (ERRs) and RTs in each task, ANOVAs included Group (MUS vs. NM) as a between-subject factor. Additional within-subject factors were Task (voicing vs. vowel length vs. aspiration) in the phonological categorization task and Condition (match vs. mismatch or related vs. unrelated, respectively) in the matching and semantic tasks.

ERPs in the phonological categorization task were analyzed by computing N100 maximum amplitudes in the 90–160 ms latency band. During the word learning phase 1, as well as in the matching and semantic tasks, ERPs were analyzed by computing the mean amplitudes of the N200 and N400 components in specific latency bands defined from visual inspection of the traces and from previous results in the literature ANOVAs included Group (MUS vs. NM) as a between-subject factor, Laterality (left: F3, C3, P3; midline: Fz, Cz, Pz; right: F4, C4, P4), and Anterior/Posterior (frontal: F3, Fz, F4; central: C3, Cz, C4; parietal: P3, Pz, P4) as within-subject factors. Additional within-subjects factors were Task (voicing vs. vowel length vs. aspiration) in the phonological categorization task, Block (1 vs. 2 vs. 3 vs. 4) in the word learning phase 1, and Condition (match vs. mismatch or related vs. unrelated, respectively) in the matching and semantic tasks. *Post hoc* Tukey tests (reducing the probability of Type I errors) were used to determine the origin of significant main effects and interactions. Finally, to examine the relationship between musical aptitude (i.e., ERRs in the musicality tasks) and word learning (i.e., ERRs in the semantic task), a linear regression model was fitted, with level of word learning as the dependent variable and level of musical aptitude as the predictor.

## Results

### Screening Measures

#### Cognitive and Language Abilities

Results of univariate ANOVAs showed no significant group differences in auditory short-term memory (*F*_(1,21)_ = 0.03, *p* = 0.88, *η*^2^ = 0.001), visual attention (*F*_(1,21)_ = 0.22, *p* = 0.65, *η*^2^ = 0.01), word reading (*F*_(1,21)_ = 2.42, *p* = 0.13, *η*^2^ = 0.10), phoneme fusion (*F*_(1,21)_ = 0.65, *p* = 0.43, *η*^2^ = 0.03), or nonverbal intelligence (PM47, *F*_(1,21)_ = 3.71, *p* = 0.07, *η*^2^ = 0.15).

#### Musical Aptitude

Results of two-way ANOVAs (i.e., 2 Groups × 2 Tasks) showed that MUS made fewer errors (19.3%) than NM (30.2%; main effect of Group: *F*_(1,21)_ = 5.76, *p* = 0.03). In addition, all children performed better on the rhythmic (18.6%) than the melodic task (31.0%; main effect of Task: *F*_(1,21)_ = 15.44, *p* < 0.001) with no Group by Task interaction (*F*_(1,21)_ = 0.01, *p* = 0.93).

### Experimental Tasks

#### Phonological Categorization

##### Behavioral data

**Figure 2:**
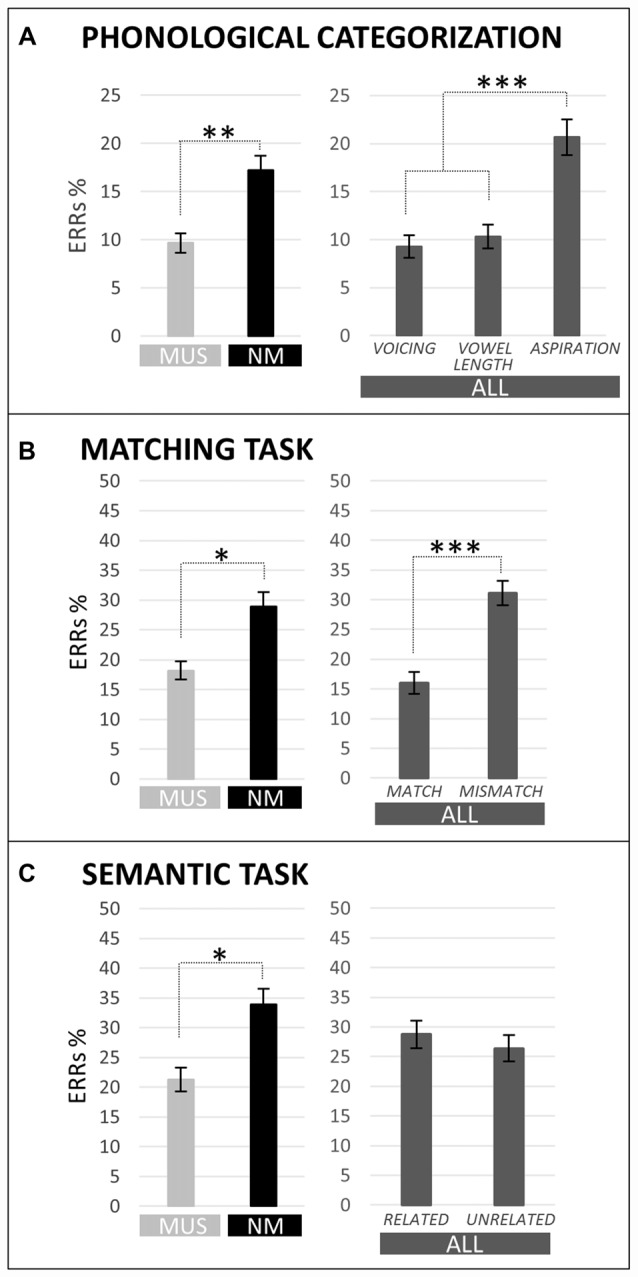
**Percentages of error (ERRs) are compared for children with music training (MUS) in light gray and for children without music training (NM) in black in the different tasks (main effect of group).** Moreover, ERRs in the different tasks are also shown averaged across children (ALL; main effect of condition) in dark gray. For the phonological categorization tasks **(A)**, results are illustrated for voicing, vowel length and aspiration. For the matching task **(B)**, results are illustrated for Match and Mismatch words. For the semantic task **(C)**, results are illustrated for semantically Related and Unrelated words. Level of significance is indicated by stars with *p < 0.05, **p < 0.01 and ***p < 0.001.

Results of two-way ANOVAs [i.e., 2 Groups × 3 Tasks (voicing vs. vowel length vs. aspiration)] showed that MUS (9.7%) made overall fewer errors than NM (17.2%; main effect of Group: *F*_(1,21)_ = 7.17, *p* = 0.01; see Figure 2A). Furthermore, across children, the error rate was lower in the voicing and vowel length tasks (9.3% and 10.3%, respectively) than in the aspiration task (20.7%; Tukey, both *p*s < 0.001; main effect of Task: *F*_(2,42)_ = 11.54, *p* < 0.001; no Group by Task interaction).

##### Electrophysiological data

**Figure 3:**
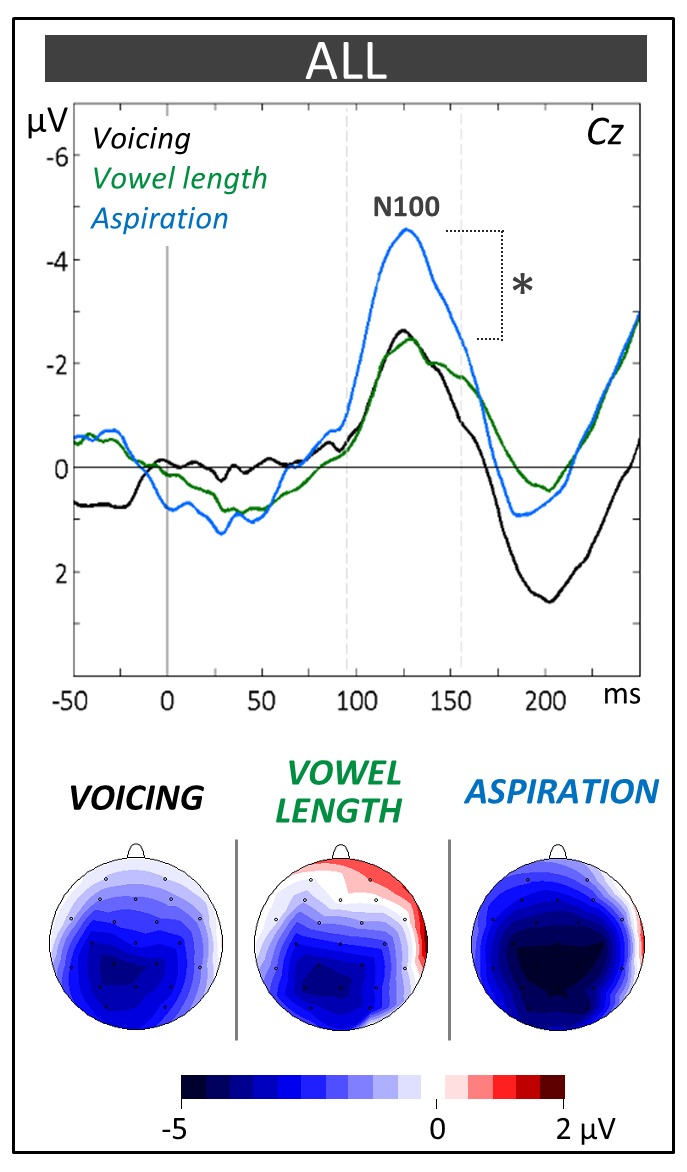
**Phonological categorization tasks**. N100 components at the Central (Cz) electrode and topographic voltage distribution maps are compared between tasks averaged across children (ALL). Voltage values of topographic maps are scaled from –5 μV to +2 μV. Mean number of trials contributing to the averages (*n* = 1228). In this and subsequent ERPs figures, time in milliseconds is in abscissa and the amplitude of the effects in microvolt is in ordinate. Time zero corresponds to word onset and negativity is plotted upwards. Latency windows for statistical analyses are indicated with gray dotted lines and the level of significance is indicated by stars with *p < 0.05, **p < 0.01 and ***p < 0.001.

The N100 component (90–160 ms) was evaluated by means of a 2 × 3 × 3 × 3 ANOVA (i.e., 2 Groups × 3 Tasks × 3 Laterality positions (left vs. midline vs. right) × 3 Anterior/Posterior positions [frontal vs. central vs. parietal]). As can be seen on Figure 3, the N100 amplitude was larger in the aspiration task (−6.01 μV, SD = 1.96) compared to the other tasks (−4.42 μV, SD = 1.54; Tukey, *p* = 0.03; main effect of Task: *F*_(2,42)_ = 4.01, *p* = 0.03). No significant main effect of Group (*F*_(1,21)_ = 1.02, *p* = 0.32) or interaction including the Group factor was obtained (Group × Task × Anterior/Posterior interaction: *F*_(4,84)_ = 1.87, *p* = 0.12).

#### Word Learning Phase 1

##### Electrophysiological data

**Figure 4:**
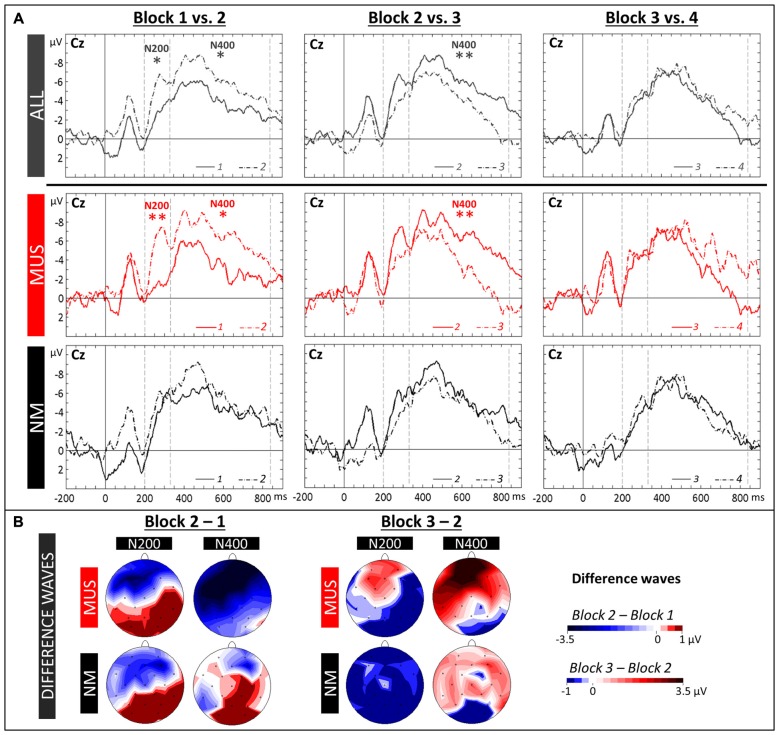
**Word learning phase 1. (A)** ERPs recorded at the Central (Cz) electrode in the four Blocks are overlapped separately for Block 1 and 2 (left column), Block 2 and 3 (central column) and Block 3 and 4 (right column). Recordings are presented averaged across all children (ALL, dark gray lines) and for musician (MUS: red lines) and non-musician children (NM: black lines). Mean number of trials contributing to the averages (ALL = 607, MUS = 317, NM = 290). **(B)** Topographic voltage distribution maps of the differences between two blocks (2–1: Block 2 minus Block 1, and 3–2: Block 3 minus Block 2) are illustrated for the N200 and N400, separately for MUS and for NM. Voltage values are scaled from –3.5 μV to +1.0 μV and –1.0 μV to +3.5 μV, respectively.

The N400 (330–860 ms) as well as the N200 (200–330 ms) were evaluated by means of 2 × 4 × 3 × 3 ANOVAs (i.e., 2 Groups × 4 Blocks [1 vs. 2 vs. 3 vs. 4] × 3 Laterality × 3 Anterior/Posterior positions). For all children and in line with previous results, the N400 component was larger over frontal (−4.89 μV, SD = 1.52) and central (−4.62 μV, SD = 1.23) sites compared to parietal sites (−1.87 μV, SD = 1.32; Tukey, both *p*s < 0.001; main effect of Anterior/Posterior: *F*_(2,42)_ = 33.59, *p* < 0.001). In addition, while the Group × Block interaction was only marginally significant (*F*_(3,63)_ = 2.17, *p* = 0.10), both the increase in N400 from Block 1 to Block 2 and the decrease from Block 2 to Block 3 were significant for MUS (Block 1: −2.79 μV, SD = 1.66; Block 2: −5.46 μV, SD = 2.10; Block 3: −2.23 μV, SD = 2.04; Tukey, 1 vs. 2: *p* = 0.02, and 2 vs. 3: *p* = 0.005; main effect of Block: *F*_(3,33)_ = 5.34, *p* = 0.004) but not for NM (main effect of Block: *F*_(3,30)_ = 1.13, *p* = 0.35; see Figures 4A,B for topographic distributions of effects). Similarly, for the N200, the increase from Block 1 to Block 2 was significant in MUS but not in NM (MUS: Block 1: −1.64 μV, SD = 2.35; Block 2: −4.68 μV, SD = 1.98; Tukey, 1 vs. 2: *p* = 0.01; main effect of Block: *F*_(3,33)_ = 3.82, *p* = 0.02; and NM: main effect of Block: *F*_(3,30)_ = 0.19, *p* = 0.90).

#### Word Learning Phase 2

##### Behavioral data

No significant group differences were found on ERRs (MUS: 16.0% and NM: 20.2%; main effect of Group: *F*_(1,21)_ = 1.12, *p* = 0.30).

#### Matching Task

##### Behavioral data

Results of two-way ANOVAs [i.e., 2 Groups × 2 Conditions (match vs. mismatch)] showed that MUS (18.2%) made significantly fewer errors than NM (28.9%; main effect of Group: *F*_(1,21)_ = 5.54, *p* = 0.03; see Figure 2B) and that all children made fewer errors for match (16.0%) than for mismatch words (31.1%; main effect of Condition: *F*_(1,21)_ = 22.66, *p* < 0.001). The Group by Condition interaction was not significant (*F*_(1,21)_ = 1.82, *p* = 0.19). No significant between-group differences and no Group by Condition interaction were found on RTs (*F*_(1,21)_ = 0.13, *p* = 0.72, and *F*_(1,21)_ = 0.32, *p* = 0.58, respectively) but, in line with error rates, RTs were faster for match (1284 ms) than for mismatch words (1461 ms; main effect of Condition: *F*_(1,21)_ = 35.13, *p* < 0.001).

##### Electrophysiological data

**Figure 5:**
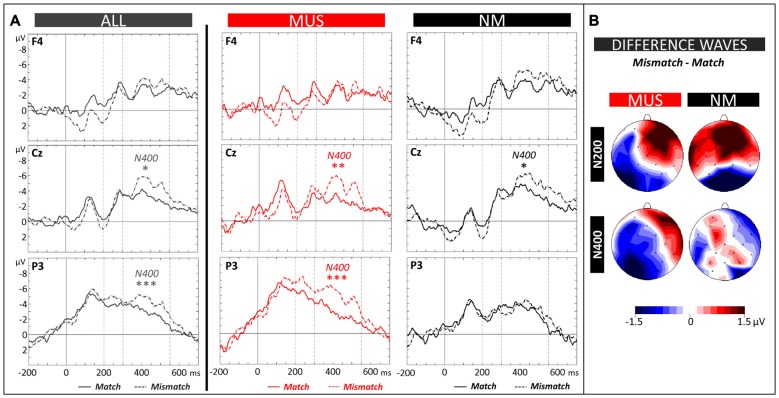
**Matching task. (A)** ERPs recorded at representative electrodes (right frontal (F4), central (Cz) and left parietal (P3)) are overlapped for Match (solid lines) and Mismatch words (dotted lines), averaged across all children (ALL, dark gray lines) and for musician (MUS, red lines) and non-musician children (NM, black lines). Mean number of trials contributing to the averages (ALL = 1215, MUS = 634, NM = 581). **(B)** Topographic voltage distribution maps of the differences between conditions (Mismatch minus Match) are illustrated for the N200 and N400 and for MUS and NM. Voltage values are scaled from –1.5 μV to +1.5 μV.

The N400 (300–550 ms) and the N200 (200–300 ms) components were evaluated by means of 2 × 2 × 3 × 3 ANOVAs (2 Groups × 2 Conditions × 3 Laterality × 3 Anterior/Posterior positions). Analysis of the N400 revealed a significant Group × Laterality × Anterior/Posterior interaction (*F*_(4,84)_ = 2.44, *p* = 0.05). Results of separate group analyses showed larger N400 amplitude in MUS for mismatch (−4.15 μV, SD = 1.95) compared to match words across all scalp sites (−2.75 μV, SD = 2.19; main effect of Condition: *F*_(1,11)_ = 5.08, *p* = 0.05; see Figures 5A,B). In NM, the N400 effect was localized over central electrodes (mismatch: −5.81 μV, SD = 1.15 and match words: −4.62 μV, SD = 0.89) with slightly larger differences over the right than left hemisphere (Condition × Laterality × Anterior/Posterior interaction: *F*_(4,40)_ = 2.79, *p* = 0.04; see Figures 5A,B). Finally, analyses of the N200 showed larger amplitude in MUS compared to NM over the left hemisphere (MUS: −3.73 μV, SD = 1.50 and NM: −1.57 μV, SD = 1.57; Group × Laterality: *F*_(2,42)_ = 3.20, *p* = 0.05) but no interaction involving the factor Condition.

#### Semantic Task

##### Behavioral data

Results of two-way ANOVAs [i.e., 2 Groups × 2 Conditions (related vs. unrelated)] showed that MUS (23.1%) made significantly fewer errors than NM (33.9%; main effect of Group: *F*_(1,21)_ = 4.50, *p* = 0.05; see Figure 2C) and that all children made as many errors to related (29.7%) than to unrelated words (27.2%; main effect of Condition: *F*_(1,21)_ = 0.39, *p* = 0.54). The Group by Condition interaction was not significant on ERRs (*F*_(1,21)_ = 0.20, *p* = 0.66). RTs were faster to semantically related (1996 ms) than to unrelated words (2296 ms; main effect of Condition: *F*_(1,21)_ = 58.42, *p* < 0.001) with no significant between-group differences and no Group by Condition interaction (*F*_(1,21)_ = 0.29, *p* = 0.60, and *F*_(1,21)_ = 0.32, *p* = 0.58, respectively).

##### Electrophysiological data

**Figure 6:**
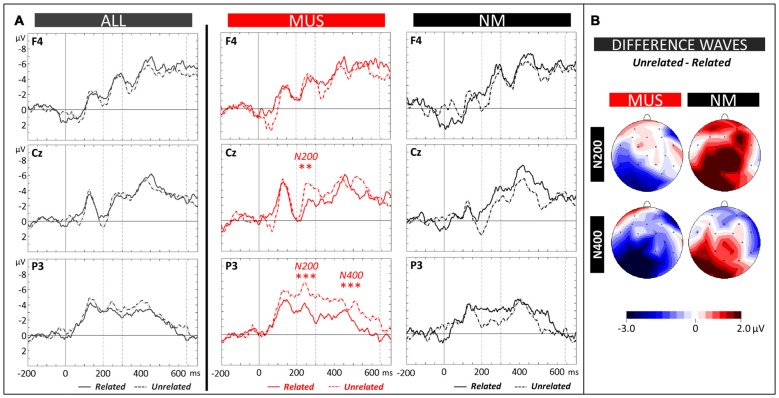
**Semantic task. (A)** ERPs recorded at representative electrodes (right frontal (F4), central (Cz) and left parietal (P3)) are overlapped for Related (solid lines) and Unrelated words (dotted lines), averaged across all children (ALL, dark gray lines) and for musician (MUS, red lines) and non-musician children (NM, black lines). Mean number of trials contributing to the averages (ALL = 730, MUS = 380, NM = 350). **(B)** Topographic voltage distribution maps of the differences between conditions (Unrelated minus Related) are illustrated for the N200 and N400 and for MUS and NM. Voltage values are scaled from –3.0 μV to +2.0 μV.

The N400 (300–640 ms) and the N200 (200–300 ms) components were evaluated by means of 2 × 2 × 3 × 3 ANOVAs (2 Groups × 2 Conditions × 3 Laterality × 3 Anterior/Posterior positions). Analysis of the N400 revealed a significant Group × Condition × Anterior/Posterior interaction (*F*_(2,42)_ = 2.64, *p* = 0.05). Results of separate analyses (see Figure 6A for MUS and NM, and Figure 6B for topographic distributions of effects) showed that for MUS, the N400 over parietal regions was larger to unrelated (−2.72 μV, SD = 1.32) than to related words (−1.35 μV, SD = 1.09; Tukey, *p* = 0.03; Condition × Anterior/Posterior interaction: *F*_(2,22)_ = 3.79, *p* = 0.04). This effect was not significant for NM (Condition × Anterior/Posterior interaction: *F*_(2,20)_ = 1.07, *p* = 0.36). However, as previously found in NM adults, the N400 effect was reversed with larger N400 for related (−5.03 μV, SD = 0.81) than for unrelated words at left central sites (−3.38 μV, SD = 0.78; Tukey, *p* = 0.04; Condition × Laterality × Anterior/Posterior interaction: *F*_(4,40)_ = 2.20, *p* = 0.09).

Similarly, analysis of the N200 revealed significant Group × Condition and Group × Condition × Anterior/Posterior interactions (*F*_(2,21)_ = 5.21, *p* = 0.03 and *F*_(2,42)_ = 5.39, *p* = 0.008). Results of separate analysis showed that for MUS the N200 over central and parietal regions was larger to unrelated (−4.48 μV, SD = 1.37 and −4.90 μV, SD = 1.10, respectively) than to related words (−3.00 μV, SD = 1.11 and −2.57 μV, SD = 0.69; Tukey, *p* = 0.05 and < 0.001, respectively; Condition × Anterior/Posterior interaction: *F*_(2,22)_ = 3.74, *p* = 0.04) with no significant differences for NM (main effect of Condition: *F*_(1,10)_ = 1.46, *p* = 0.25, and Condition × Anterior/Posterior interaction: *F*_(2,20)_ = 0.40, *p* = 0.67).

### Relationship between Musical Aptitude and Word Learning

**Figure 7:**
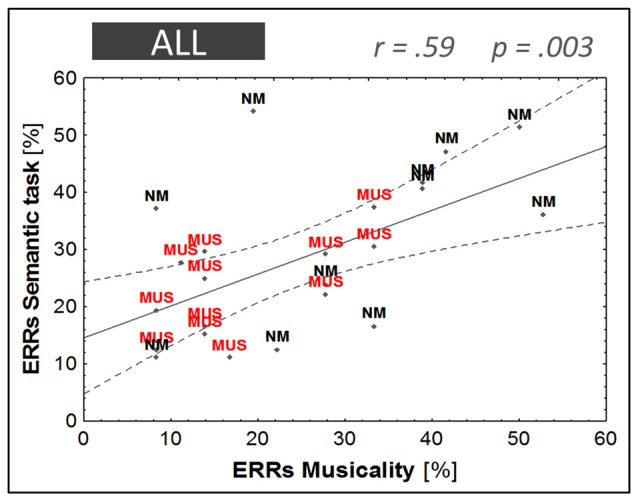
**Linear regression model**. Musical aptitude (ERRs in the musicality task) against word learning performance (ERRs in the semantic task). Children with music training are illustrated in red (MUS) and children without music training in black (NM).

A highly significant correlation was found between musical aptitude and word learning (*R*^2^ = 0.31, *F*_(1,21)_ = 10.94, *p* = 0.003), which reflected the fact that children with fewer errors in the musical aptitude task (i.e., musically-trained children) achieved higher levels of word learning (i.e., fewer errors in the semantic task; see Figure 7). As screening measures showed a trend towards group differences on nonverbal intelligence (PM47, *p* = 0.07), and as age differences although not significant (*p* = 0.19) may influence word learning performance, two separate partial correlations were computed controlling for these variables. In both cases, the partial correlation between musical aptitudes and word learning remained highly significant when controlling for PM47 (*r* = 0.51, *p* = 0.01) or age (*r* = 0.51, *p* = 0.02).

## Discussion

This series of experiments revealed three main findings. First, ERPs recorded in the word learning phase showed that the temporal dynamics of novel word learning, as reflected by significant modulations of N200 and FN400 amplitudes after only a few minutes of picture-word associative learning, was faster in children with music training than in control children. Second, while all children were able to learn the meaning of new words, music training was associated with more efficient learning of picture-word associations as reflected by both behavioral and electrophysiological data in the matching and semantic tasks. Finally, a fronto-parietal network was involved in word learning with a shift of the distribution of the N200 and N400 components from frontal regions in the learning phase to parietal regions during the test phase (matching and semantic tasks). These findings are discussed below.

### Fast Brain Plasticity in the Word Learning Phase

Recording ERPs during the four blocks of the learning phase allowed us to precisely follow the temporal dynamics of the learning process. ERPs averaged across all children clearly showed that large changes in brain activity occurred very rapidly during the acquisition of word meaning (see Figure 4A). As hypothesized based on previous results in adults (Mestres-Missé et al., 2007; Borovsky et al., 2012; Dittinger et al., 2016; François et al., 2017) and in infants (Torkildsen et al., 2006; Friedrich and Friederici, 2008), results showed an increased long-lasting negativity from Block 1 to Block 2 over fronto-central sites, comprising both an N200 and an FN400 components, taken to reflect learning of novel picture-word associations (see Figures 4A,B, topographic maps). It is notable that the N200 component is more clearly visible than in previous experiments, possibly due to auditory rather than visual word presentation. Moreover, the overall amplitude of the N200 and FN400 components is much larger, and the FN400 component is longer-lasting, in children than in adults (Dittinger et al., 2016; see Figure 4). The differences between Block 1 and Block 2 were localized over fronto-central regions. This scalp distribution is very similar to previous results in word segmentation experiments (Cunillera et al., 2009; François et al., 2014) and is compatible with previous findings suggesting that prefrontal and temporal brain regions are associated with the maintenance of novel information in working memory (Hagoort, 2014) and with the acquisition of word meaning (Rodríguez-Fornells et al., 2009). What is most remarkable is that these amplitude modulations were observed after only 3 min of learning novel word meanings (that is after only 10 repetitions of each picture-word association), thereby showing clear evidence for fast mapping (Carey, 1978) as reflected by fast changes in brain activity. Importantly, and as previously found in adults (Dittinger et al., 2016), these effects were significant in musically-trained children but not in children without music training. Thus, in line with our hypothesis, these results showed evidence for faster encoding of novel word meaning in musically-trained children. Interestingly, and strikingly similar to previous results in word segmentation experiments (Cunillera et al., 2009; François et al., 2014), FN400 amplitude was already decreased from Block 2 to Block 3 (i.e., after 3–4 min), possibly due to repetition effects (Rugg, 1985) that contribute to learning. Cunillera et al. (2009) interpret their similar findings in light of the time-dependent hypothesis (Raichle et al., 1994; Poldrack et al., 1999) following which increased activation (as reflected by the FN400) is only found during the initial learning period and quickly decreases when words have been identified, or, as in our experiment, when meaning has been attached to the auditory word-form. Finally, no differences were found between Block 3 and Block 4 possibly because all children had reached a learning threshold.

Turning to the N200 component, and in contrast to what was previously found in adult non-musicians (Dittinger et al., 2016), the differences between Block 1 and Block 2 were not significant in children with no specific music training. Insofar as the N200 reflects categorization processes (Friedrich and Friederici, 2008), it may be that these children had not yet learned to categorize the correct word with the correct picture. Alternatively, and based on recent results by Du et al. (2014) showing enhanced N200 amplitude when Chinese compound words are repeated in priming experiments, it may be that adult non-musicians were more sensitive to the repetition of words in the learning phase than children with no music training.

### Testing Novel Word Learning in the Matching and Semantic Tasks

All children were able to learn the six picture-word associations within a short learning phase (around 12 min total time for both word learning phases 1 and 2) as shown by the low percentage of errors in the active learning phase (<21% in both groups) and in the matching and semantic tasks (between 13% and 38% across groups). Importantly, the level of performance in both tasks and in both groups was above chance level (50%) and far from ceiling or floor effects thereby showing that the level of task difficulty was not too easy nor too difficult. In line with previous findings in the literature and with findings in adults using a similar design (Dittinger et al., 2016), results in the matching task showed clear matching effects with lower error rates and faster RTs to matching than to mismatching words thereby showing that all children had learned the picture-word associations presented in the word learning phase. Moreover, this learning effect generalized to new pictures in the semantic task, as revealed by faster RTs to auditory words semantically related to new pictures than to unrelated words (Meyer and Schvaneveldt, 1971; Dittinger et al., 2016). However, the semantic priming effect was not significant on error rates. While surprising, this finding possibly reflects a response bias towards rejection: when children were not certain whether pictures and words were semantically related (e.g., “honey” and “bear”), they tended to respond that they were unrelated. This interpretation is in line with the adult results showing that participants made significantly fewer errors for unrelated than for related words.

Finally, and perhaps most importantly, while the matching (on both errors and RTs) and semantic priming effects (on RTs) were significant in both groups (no Group by Condition interaction), musically-trained children made significantly fewer errors than controls in both the matching and the semantic tasks (main effect of Group), suggesting that they had learned the meaning of novel words more efficiently than controls. Importantly, the effect of musicianship in children was very similar to what was found in adults (Dittinger et al., 2016). In the semantic task, adult musicians outperformed adult non-musicians. Moreover, the level of performance was similar in both groups (musician children [23.1%] and adults [23.6%]; non-musician children [33.9%] and adults [30.5%] thereby showing that the level of task difficulty was similar for children [learning 6 novel words] and adults [learning 9 novel words]). In the matching task, adult musicians made fewer errors than adult non-musicians but, in contrast to children, this difference did not reach significance possibly because the matching task was too easy to reveal a between-group difference in adults.

Comparison of the electrophysiological data in the matching task between children and adults also revealed interesting differences. While children without music training showed a typical N400 effect over central electrodes (N400 larger to mismatch than match words), adults without music training showed a reversed N400 effect over frontal electrodes (see Figure 5 of Dittinger et al., 2016) that we interpreted as showing that they had not yet fully integrated the meaning of novel words into pre-existing semantic networks. Following this interpretation, non-musician children, by showing typical N400 effects, were faster in integrating the meaning of novel words than non-musician adults. However, this speculation needs further support to be convincing since adult non-musicians performed as well as adult musicians in the matching task but children with music training outperformed control children. Finally, as in the word learning phase, the N200 effect was significant in adults but not in children with no music training, again possibly because adults were more sensitive to word repetition (Du et al., 2014) than children. Maybe more interestingly, children without music training showed an N400 effect without an N200 effect in the matching task, thereby supporting the hypothesis that both components reflect independent processes (e.g., Du et al., 2014; see Hofmann and Jacobs, 2014; for a detailed discussion of this issue).

Turning to the semantic task and in line with the behavioral results showing that children with music training learned the meaning of novel words more efficiently than control children, N200 and N400 amplitudes were significantly larger for unrelated than for related words over parietal regions in children with music training but not in non-musician children (see Figure 6). Again, these results are very similar to previous results in adults (Dittinger et al., 2016) showing significant N200 and N400 semantic priming effects over parietal regions in adult musicians but not in non-musicians. Only the N200 effect was significant in adult non-musicians, again pointing to the independance of these two components (Du et al., 2014). By contrast, it is striking that, similar to adult non-musician results in the matching task, reversed N400 effects (larger N400 to related than to unrelated words) were found in the semantic task, both in children without music training, over left central sites (see Figure 6B) and in non-musician adults, over frontal sites (see Figure 6 of Dittinger et al., 2016). Below we propose an interpretation of these surprising results that showed up in two independent samples.

Results in the word learning literature have shown that the N400 is larger for semantically unrelated than for related words in both lexical decision tasks (Borovsky et al., 2012) and semantic priming experiments (Mestres-Missé et al., 2007). This is taken as evidence that novel words are processed differently based on previously learned associations and that, with training, the meaning of novel words is rapidly integrated into semantic memory networks (Mestres-Missé et al., 2007; Batterink and Neville, 2011; Borovsky et al., 2012). Based on this interpretation, the different N400 effects for children with and without music training in the semantic task suggest that while musically-trained children had already integrated the meaning of the novel words into semantic memory, as reflected by typical N400 effects, this was not yet the case for control children (reversed N400 effects). In other words, while all children were able to retrieve the specific picture-word associations that were stored in episodic memory during the word learning phase, as reflected by typical N400 effects in the matching task, generalization of learning as seen through priming effects from new pictures semantically related to the novel words could possibly take longer for control children than for musically-trained children. In sum, differences between musically-trained and untrained participants (both children and adults, Dittinger et al., 2016) were larger when the task required retrieving general information from semantic memory in the semantic task than retrieving specific picture-word associations in the matching task.

Finally, in contrast to the frontally-distributed N400 component during the early stages of learning discussed above, the N400 effect in the test phase was clearly centro-parietally distributed. Thus, when the meaning of words was already learned, as in the matching and semantic tasks (see Figures 5B, 6B), and as in typical N400 experiments with known words (Kutas et al., 1988), the N400 showed a more parietal scalp distribution that possibly reflects access to the meaning of words already stored in semantic memory or the integration of novel words meaning in existing semantic networks (Batterink and Neville, 2011). In sum, by recording ERPs both in the word learning phase and in the matching and semantic tasks from the same participants, we found a clear fronto-parietal shift in N400 scalp distribution with learning (compare Figures 4, 5, 6). Importantly, this shift in N400 distribution from the acquisition to the consolidation of novel word meaning was also found in adults (Batterink and Neville, 2011; Dittinger et al., 2016).

### The Cascade and Multi-Dimensional Interpretations

We previously proposed two complementary bottom-up and top-down interpretations to account for the advantage of musician compared to non-musician adults in novel word learning (Dittinger et al., 2016). Following the “cascade” interpretation (bottom-up), increased auditory sensitivity is the driving force behind enhanced word learning in musicians. According to this view, enhanced auditory perception and attention in musicians (Kraus and Chandrasekaran, 2010; Besson et al., 2011; Strait et al., 2015) allow one to build clear and stable phonological representations (Anvari et al., 2002; Corrigall and Trainor, 2011) that are more easily discriminable and consequently easier to associate with specific meanings and to store in semantic memory. Previous reports provided clear evidence that music training improves sensitivity of auditory-related brain regions (Schneider et al., 2002; Elmer et al., 2013; Kühnis et al., 2014) and fosters the ability to focus and maintain attention on auditory stimuli (Magne et al., 2006; Moreno et al., 2009; Tervaniemi et al., 2009; Strait et al., 2010, 2015; Corrigall and Trainor, 2011).

In line with these results, the level of performance in the three phonological categorization tasks (voicing, vowel length and aspiration) was significantly higher in musically-trained children than in controls (see Figure 2A). This supports the hypothesis that music training is associated with clearer and more stable phonological representations. This, in turn, may facilitate the learning of new picture-word associations in the word learning phase. However, independently of music training, the N100 amplitude was largest to the unfamiliar, non-native aspiration contrast (see Figure 3). This result differs from previous ones in adults showing larger N100s to the aspiration contrast only in professional musicians (Dittinger et al., 2016). It may be that the differential sensitivity to familiar and unfamiliar phonetic contrasts decreases from childhood to adulthood and that music training helps to maintain this sensitivity. This interpretation needs to be further tested in future experiments.

Following the multi-dimensional interpretation, music training not only improves auditory sensitivity but also other functions that are relevant for novel word learning. For instance, there is evidence that music training enhances short-term memory (Ho et al., 2003; George and Coch, 2011) and executive functions (Pallesen et al., 2010; Moreno et al., 2011; Rogalsky et al., 2011; Zuk et al., 2014). In line with this interpretation, the present results showed that music training influenced associative learning and semantic integration as reflected by larger modulations of the N200 and N400 components in the matching and semantic tasks (McLaughlin et al., 2004; Perfetti et al., 2005; Mestres-Missé et al., 2007). Moreover, there is also evidence from at least one longitudinal intervention study that 1 year of music training is enough to enhance verbal and performance IQ as compared to drama lessons (Schellenberg, 2004). Consistent with these findings, musically-trained children in our study showed a trend for higher nonverbal IQs (as measured with the PM47, *p* = 0.07) than controls. It is thus possible that children with music training performed better in the matching and semantic tasks not because music training enhanced auditory perception or different aspects of language processing but because, in general, increased cognitive abilities improved word learning (Banai and Ahissar, 2013; Zatorre, 2013). This is a difficult issue. One could indeed try to match children’s level of performance on several cognitive abilities (e.g., working and short-term memory, general intelligence). However, this might result in a selection bias against musically-trained children if superior cognitive abilities were a direct consequence of music training. Coming back to our results, it is notable that children performing higher in the musicality tests also performed higher in the most difficult semantic task (see Figure 7), and that this correlation remained highly significant also when controlling for the influence of nonverbal general intelligence. Thus, while music training is likely to influence high level cognitive functions that could facilitate word learning, the results found in the present experiment do not seem to be mediated by nonverbal intelligence. In sum, facilitated word learning in children with music training probably results from the strong interplay between improved auditory perception and higher cognitive functions so that the cascade and multidimensional interpretations are best considered as complementary.

## Conclusion

Our results showed that all children were able to learn the meaning of novel words and that, similar to previous results found in adults (Batterink and Neville, 2011; Dittinger et al., 2016) word learning was associated with a fronto-parietal shift of the topographical distribution of the N400 and N200 components that developed with learning. Importantly, a few years of music training (4.5 years on average) was found to positively correlate with word learning: musically-trained children performed higher than controls in both the matching and the semantic tasks and the electrophysiological markers of word leaning (the N200 and N400 effects) were larger in children with than without music training. To our knowledge, this is the first report of a relationship between music training and the semantic aspects of language processing in children. Importantly, these results extend previous findings showing that music training enhanced phonological awareness, reading development, word comprehension and syntactic processing (Anvari et al., 2002; Jentschke and Koelsch, 2009; Corrigall and Trainor, 2011). These results also support the hypothesis that second language learning is facilitated by musical training (Slevc, 2012; Chobert and Besson, 2013; Moreno et al., 2015) and taken together they provide strong evidence for the importance of music classes in primary school.

### Limitations and Perspectives

The first limitation of the present study is the small number of children in each group. Although we tested a relatively large group of 32 children with the aim of having 16 participants in each group, several children had to be discarded for technical reasons. Nevertheless, two main arguments support the robustness of our findings. First, even with a small sample size, the effects of main interest were significant (and therefore statistically valid, Friston, 2012) in musically-trained children and not significant in control children, thereby showing clear between-group differences. Second, as discussed above, the main effects found for children in the different experiments described here are remarkably similar to those previously found using a very similar paradigm with musician and non-musician adults (Dittinger et al., 2016). Thus, the correlation between music training and better novel word learning (both in behavior and ERPs) was replicated in two independent samples of participants.

The second limitation is that, while we would like to attribute the reported differences between musically-trained and untrained children to music training, the present experiment does not allow to rule out that differences other than music training accounted for the observed between-group differences. The only way to demonstrate the causal role of music training is to conduct a longitudinal study with non-musician children trained with music and to compare results with another group of non-musician children trained with an equally interesting non-musical activity. However, before conducting such longitudinal studies to ascertain the origins of the differences, it is first of primary importance to demonstrate differences between musically-trained and control children in novel word learning, and this was the aim of the present study.

Finally, the series of experiments used in this paradigm allowed us to test for auditory perception of linguistic and non-linguistic sounds (musicality tests), for auditory attention and for associative and semantic memory. Thus, an interesting perspective would also be to use this paradigm as a diagnostic tool to determine which specific computations and cognitive functions are impaired in children with learning difficulties or in patients with degenerative disorders.

## Author Contributions

MB, JCZ and JC designed and supervised the research; JC collected EEG data and ED analyzed the EEG data; MB and ED wrote the manuscript, and JCZ and JC contributed to the manuscript.

## Conflict of Interest Statement

The authors declare that the research was conducted in the absence of any commercial or financial relationships that could be construed as a potential conflict of interest.

## References

[B200] Anvari S. H. Trainor L. J. Woodside J. Levy B. A. (2002). Relations among musical skills, phonological processing, and early reading ability in preschool children. J. Exp. Child Psychol. 83, 111–130. 10.1016/S0022-0965(02)00124-8 12408958

[B1] Asaridou S. S. McQueen J. M. (2013). Speech and music shape the listening brain: evidence for shared domain-general mechanisms. Front. Psychol. 4:321. 10.3389/fpsyg.2013.00321 23761776 PMC3671174

[B2] Banai K. Ahissar M. (2013). Musical experience, auditory perception and reading-related skills in children. PLoS One 8:e75876. 10.1371/journal.pone.0075876 24086654 PMC3782483

[B3] Batterink L. Neville H. (2011). Implicit and explicit mechanisms of word learning in a narrative context: an event-related potential study. J. Cogn. Neurosci. 23, 3181–3196. 10.1162/jocn_a_00013 21452941 PMC3129368

[B4] Besson M. Chobert J. Marie C. (2011). Transfer of training between music and speech: common processing, attention and memory. Front. Psychol. 2:94. 10.3389/fpsyg.2011.00094 21738519 PMC3125524

[B5] Bidelman G. M. Hutka S. Moreno S. (2013). Tone language speakers and musicians share enhanced perceptual and cognitive abilities for musical pitch: evidence for bidirectionality between the domains of language and music. PLoS One 8:e60676. 10.1371/journal.pone.0060676 23565267 PMC3614545

[B6] Bidelman G. M. Weiss M. W. Moreno S. Alain C. (2014). Coordinated plasticity in brainstem and auditory cortex contributes to enhanced categorical speech perception in musicians. Eur. J. Neurosci. 40, 2662–2673. 10.1111/ejn.12627 24890664

[B7] Boersma P. Weenink D. (2011). “Praat: Doing Phonetics by Computer (computer program)”. Available online at: www.praat.org

[B8] Borgström K. Torkildsen J. V. K. Lindgren M. (2015). Substantial gains in word learning ability between 20 and 24 months: a longitudinal ERP study. Brain Lang. 149, 33–45. 10.1016/j.bandl.2015.07.002 26185047

[B9] Borovsky A. Elman J. L. Kutas M. (2012). Once is enough: N400 indexes semantic integration of novel word meanings from a single exposure in context. Lang. Learn. Dev. 8, 278–302. 10.1080/15475441.2011.614893 23125559 PMC3484686

[B10] Borovsky A. Kutas M. Elman J. (2010). Learning to use words: Event-related potentials index single-shot contextual word learning. Cognition 116, 289–296. 10.1016/j.cognition.2010.05.004 20621846 PMC2904319

[B11] Carey S. (1978). “The child as word learner,” in Linguistic Theory and Psychological Reality, eds Halle M. Bresnan J. Miller G. A., (Cambridge, MA: The MIT Press), 264–293.

[B12] Chobert J. Besson M. (2013). Musical expertise and second language learning. Brain Sci. 3, 923–940. 10.3390/brainsci3020923 24961431 PMC4061852

[B13] Chobert J. François C. Velay J.-L. Besson M. (2014). Twelve months of active musical training in 8- to 10-year-old children enhances the preattentive processing of syllabic duration and voice onset time. Cereb. Cortex 24, 956–967. 10.1093/cercor/bhs377 23236208

[B14] Chobert J. Marie C. François C. Schön D. Besson M. (2011). Enhanced passive and active processing of syllables in musician children. J. Cogn. Neurosci. 23, 3874–3887. 10.1162/jocn_a_00088 21736456

[B15] Corrigall K. A. Trainor L. J. (2011). Associations between length of music training and reading skills in children. Music Percept. 29, 147–155. 10.1525/mp.2011.29.2.147

[B16] Cunillera T. Càmara E. Toro J. M. Marco-Pallares J. Sebastián-Galles N. Ortiz H.. (2009). Time course and functional neuroanatomy of speech segmentation in adults. Neuroimage 48, 541–553. 10.1016/j.neuroimage.2009.06.069 19580874

[B17] Davis M. H. Gaskell M. G. (2009). A complementary systems account of word learning: neural and behavioral evidence. Philos. Trans. R. Soc. Lond. B Biol. Sci. 364, 3773–3800. 10.1098/rstb.2009.0111 19933145 PMC2846311

[B18] De Boer T. Scott L. S. Nelson C. A. (2005). “ERPs in developmental populations,” in Event-Related Potentials: A Methods Handbook, ed. Handy T. (Cambridge, MA: The MIT Press), 263–297.

[B19] De Diego Balaguer R. Toro J. M. Rodriguez-Fornells A. Bachoud-Lévi A.-C. (2007). Different neurophysiological mechanisms underlying word and rule extraction from speech. PLoS One 2:e1175. 10.1371/journal.pone.0001175 18000546 PMC2063512

[B20] Delogu F. Lampis G. Olivetti Belardinelli M. (2006). Music-to-language transfer effect: may melodic ability improve learning of tonal languages by native nontonal speakers? Cogn. Process. 7, 203–207. 10.1007/s10339-006-0146-7 16897065

[B21] Dittinger E. Barbaroux M. D’Imperio M. Jäncke L. Elmer S. Besson M. (2016). Professional music training and novel word learning: from faster semantic encoding to longer-lasting word representations. J. Cogn. Neurosci. 28, 1584–1602. 10.1162/jocn_a_00997 27315272

[B22] Dobel C. Junghöfer M. Breitenstein C. Klauke B. Knecht S. Pantev C.. (2010). New names for known things: on the association of novel word forms with existing semantic information. J. Cogn. Neurosci. 22, 1251–1261. 10.1162/jocn.2009.21297 19583468

[B23] Dobel C. Lagemann L. Zwitserlood P. (2009). Non-native phonemes in adult word learning: evidence from the N400m. Philos. Trans. R. Soc. Lond. B Biol. Sci. 364, 3697–3709. 10.1098/rstb.2009.0158 19933141 PMC2846316

[B24] Du Y. Zhang Q. Zhang J. X. (2014). Does N200 reflect semantic processing?—An ERP study on Chinese visual word recognition. PLoS One 9:e90794. 10.1371/journal.pone.0090794 24622389 PMC3951240

[B25] Dumay N. Gaskell M. G. (2007). Sleep-associated changes in the mental representation of spoken words. Psychol. Sci. 18, 35–39. 10.1111/j.1467-9280.2007.01845.x 17362375

[B26] Elmer S. Hänggi J. Meyer M. Jäncke L. (2013). Increased cortical surface area of the left planum temporale in musicians facilitates the categorization of phonetic and temporal speech sounds. Cortex 49, 2812–2821. 10.1016/j.cortex.2013.03.007 23628644

[B27] Elmer S. Klein C. Kühnis J. Liem F. Meyer M. Jäncke L. (2014). Music and language expertise influence the categorization of speech and musical sounds: behavioral and electrophysiological measurements. J. Cogn. Neurosci. 26, 2356–2369. 10.1162/jocn_a_00632 24702451

[B28] Elmer S. Meyer M. Jancke L. (2012). Neurofunctional and behavioral correlates of phonetic and temporal categorization in musically trained and untrained subjects. Cereb. Cortex 22, 650–658. 10.1093/cercor/bhr142 21680844

[B29] François C. Chobert J. Besson M. Schon D. (2013). Music training for the development of speech segmentation. Cereb. Cortex 23, 2038–2043. 10.1093/cercor/bhs180 22784606

[B30] François C. Cunillera T. Garcia E. Laine M. Rodriguez-Fornells A. (2017). Neurophysiological evidence for the interplay of speech segmentation and word-referent mapping during novel word learning. Neuropsychologia 98, 56–67. 10.1016/j.neuropsychologia.2016.10.006 27732869

[B31] François C. Jaillet F. Takerkart S. Schön D. (2014). Faster sound stream segmentation in musicians than in nonmusicians. PLoS One 9:e101340. 10.1371/journal.pone.0101340 25014068 PMC4094420

[B32] Friedrich M. Friederici A. D. (2008). Neurophysiological correlates of online word learning in 14-month-old infants. Neuroreport 19, 1757–1761. 10.1097/WNR.0b013e328318f014 18955904

[B33] Friedrich M. Wilhelm I. Born J. Friederici A. D. (2015). Generalization of word meanings during infant sleep. Nat. Commun. 6:6004. 10.1038/ncomms7004 25633407 PMC4316748

[B34] Friston K. (2012). Ten ironic rules for non-statistical reviewers. Neuroimage 61, 1300–1310. 10.1016/j.neuroimage.2012.04.018 22521475

[B35] Gandour J. Wong D. Lowe M. Dzemidzic M. Satthamnuwong N. Tong Y.. (2002). A cross-linguistic FMRI study of spectral and temporal cues underlying phonological processing. J. Cogn. Neurosci. 14, 1076–1087. 10.1162/089892902320474526 12419130

[B36] George E. M. Coch D. (2011). Music training and working memory: an ERP study. Neuropsychologia 49, 1083–1094. 10.1016/j.neuropsychologia.2011.02.001 21315092

[B37] Gordon R. L. Shivers C. M. Wieland E. A. Kotz S. A. Yoder P. J. Devin McAuley J. (2015). Musical rhythm discrimination explains individual differences in grammar skills in children. Dev. Sci. 18, 635–644. 10.1111/desc.12230 25195623

[B38] Hagoort P. (2014). Nodes and networks in the neural architecture for language: Broca’s region and beyond. Curr. Opin. Neurobiol. 28, 136–141. 10.1016/j.conb.2014.07.013 25062474

[B39] Hahne A. Eckstein K. Friederici A. D. (2004). Brain signatures of syntactic and semantic processes during children’s language development. J. Cogn. Neurosci. 16, 1302–1318. 10.1162/0898929041920504 15453981

[B40] Ho Y.-C. Cheung M.-C. Chan A. S. (2003). Music training improves verbal but not visual memory: cross-sectional and longitudinal explorations in children. Neuropsychology 17, 439–450. 10.1037/0894-4105.17.3.439 12959510

[B41] Hofmann M. J. Jacobs A. M. (2014). Interactive activation and competition models and semantic context: from behavioral to brain data. Neurosci. Biobehav. Rev. 46, 85–104. 10.1016/j.neubiorev.2014.06.011 24992217

[B42] Holcomb P. J. Coffey S. A. Neville H. J. (1992). Visual and auditory sentence processing: A developmental analysis using event-related brain potentials. Dev. Neuropsychol. 8, 203–241. 10.1080/87565649209540525

[B43] Huss M. Verney J. P. Fosker T. Mead N. Goswami U. (2011). Music, rhythm, rise time perception and developmental dyslexia: perception of musical meter predicts reading and phonology. Cortex 47, 674–689. 10.1016/j.cortex.2010.07.010 20843509

[B44] Jacquier-Roux M. Valdois S. Zorman M. (2005). Outil de Dépistage des Dyslexies. Grenoble: Cogni-Sciences.

[B45] Jasper H. (1958). The ten twenty electrode system of the international federation. Electroencephalogr. Clin. Neurophysiol. 10, 371–375. 10590970

[B46] Jentschke S. Koelsch S. (2009). Musical training modulates the development of syntax processing in children. Neuroimage 47, 735–744. 10.1016/j.neuroimage.2009.04.090 19427908

[B47] Junge C. Cutler A. Hagoort P. (2012). Electrophysiological evidence of early word learning. Neuropsychologia 50, 3702–3712. 10.1016/j.neuropsychologia.2012.10.012 23108241

[B48] Juottonen K. Revonsuo A. Lang H. (1996). Dissimilar age influences on two ERP waveforms (LPC and N400) reflecting semantic context effect. Cogn. Brain Res. 4, 99–107. 10.1016/0926-6410(96)00022-5 8883923

[B49] Korkman M. Kirk U. Kemp S. (1998). NEPSY: A Developmental Neuropsychological Assessment. San Antonio, TX: The Psychological Corporation.

[B50] Kraus N. Chandrasekaran B. (2010). Music training for the development of auditory skills. Nat. Rev. Neurosci. 11, 599–605. 10.1038/nrn2882 20648064

[B51] Kühnis J. Elmer S. Jäncke L. (2014). Auditory evoked responses in musicians during passive vowel listening are modulated by functional connectivity between bilateral auditory-related brain regions. J. Cogn. Neurosci. 26, 2750–2761. 10.1162/jocn_a_00674 24893742

[B52] Kühnis J. Elmer S. Meyer M. Jäncke L. (2013). The encoding of vowels and temporal speech cues in the auditory cortex of professional musicians: an EEG study. Neuropsychologia 51, 1608–1618. 10.1016/j.neuropsychologia.2013.04.007 23664833

[B53] Kutas M. Federmeier K. D. (2011). Thirty years and counting: finding meaning in the N400 component of the event-related brain potential (ERP). Annu. Rev. Psychol. 62, 621–647. 10.1146/annurev.psych.093008.131123 20809790 PMC4052444

[B54] Kutas M. Hillyard S. A. (1980). Reading senseless sentences: brain potentials reflect semantic incongruity. Science 207, 203–205. 10.1126/science.7350657 7350657

[B55] Kutas M. Van Petten C. Besson M. (1988). Event-related potential asymmetries during the reading of sentences. Electroencephalogr. Clin. Neurophysiol. 69, 218–233. 10.1016/0013-4694(88)90131-9 2450003

[B56] Lima C. F. Castro S. L. (2011). Speaking to the trained ear: musical expertise enhances the recognition of emotions in speech prosody. Emotion 11, 1021–1031. 10.1037/a0024521 21942696

[B57] Magne C. Schön D. Besson M. (2006). Musician children detect pitch violations in both music and language better than nonmusician children: behavioral and electrophysiological approaches. J. Cogn. Neurosci. 18, 199–211. 10.1162/089892906775783660 16494681

[B58] Marie C. Magne C. Besson M. (2011). Musicians and the metric structure of words. J. Cogn. Neurosci. 23, 294–305. 10.1162/jocn.2010.21413 20044890

[B59] Markson L. Bloom P. (1997). Evidence against a dedicated system for word learning in children. Nature 385, 813–815. 10.1038/385813a0 9039912

[B60] Marques C. Moreno S. Castro S. L. Besson M. (2007). Musicians detect pitch violation in a foreign language better than nonmusicians: behavioral and electrophysiological evidence. J. Cogn. Neurosci. 19, 1453–1463. 10.1162/jocn.2007.19.9.1453 17714007

[B61] McLaughlin J. Osterhout L. Kim A. (2004). Neural correlates of second-language word learning: minimal instruction produces rapid change. Nat. Neurosci. 7, 703–704. 10.1038/nn1264 15195094

[B62] Mestres-Missé A. Rodriguez-Fornells A. Münte T. F. (2007). Watching the brain during meaning acquisition. Cereb. Cortex 17, 1858–1866. 10.1093/cercor/bhl094 17056648

[B63] Meyer D. E. Schvaneveldt R. W. (1971). Facilitation in recognizing pairs of words: evidence of a dependance between retrieval operations. J. Exp. Psychol. 90, 227–234. 10.1037/h0031564 5134329

[B64] Moreno S. Bialystok E. Barac R. Schellenberg E. G. Cepeda N. J. Chau T. (2011). Short-term music training enhances verbal intelligence and executive function. Psychol. Sci. 22, 1425–1433. 10.1177/0956797611416999 21969312 PMC3449320

[B65] Moreno S. Lee Y. Janus M. Bialystok E. (2015). Short-term second language and music training induces lasting functional brain changes in early childhood. Child Dev. 86, 394–406. 10.1111/cdev.12297 25346534 PMC4376572

[B66] Moreno S. Marques C. Santos A. Santos M. Castro S. L. Besson M. (2009). Musical training influences linguistic abilities in 8-year-old children: more evidence for brain plasticity. Cereb. Cortex 19, 712–723. 10.1093/cercor/bhn120 18832336

[B67] Musacchia G. Sams M. Skoe E. Kraus N. (2007). Musicians have enhanced subcortical auditory and audiovisual processing of speech and music. Proc. Natl. Acad. Sci. U S A 104, 15894–15898. 10.1073/pnas.0701498104 17898180 PMC2000431

[B68] Pallesen K. J. Brattico E. Bailey C. J. Korvenoja A. Koivisto J. Gjedde A.. (2010). Cognitive control in auditory working memory is enhanced in musicians. PLoS One 5:e11120. 10.1371/journal.pone.0011120 20559545 PMC2886055

[B69] Parbery-Clark A. Tierney A. Strait D. L. Kraus N. (2012). Musicians have fine-tuned neural distinction of speech syllables. Neuroscience 219, 111–119. 10.1016/j.neuroscience.2012.05.042 22634507 PMC3402586

[B70] Patel A. D. (2008). Music, Language and The Brain. Oxford: Oxford University Press.

[B71] Peretz I. Champod A. S. Hyde K. (2003). Varieties of musical disorders. the montreal battery of evaluation of amusia. Ann. N Y Acad. Sci. 999, 58–75. 10.1196/annals.1284.006 14681118

[B72] Perfetti C. A. Wlotko E. W. Hart L. A. (2005). Word learning and individual differences in word learning reflected in event-related potentials. J. Exp. Psychol. Learn. Mem. Cogn. 31, 1281–1292. 10.1037/0278-7393.31.6.1281 16393047

[B73] Perruchet P. Poulin-Charronnat B. (2013). Challenging prior evidence for a shared syntactic processor for language and music. Psychon. Bull. Rev. 20, 310–317. 10.3758/s13423-012-0344-5 23180417

[B74] Poldrack R. A. Wagner A. D. Prull M. W. Desmond J. E. Glover G. H. Gabrieli J. D. (1999). Functional specialization for semantic and phonological processing in the left inferior prefrontal cortex. Neuroimage 10, 15–35. 10.1006/nimg.1999.0441 10385578

[B75] Raichle M. E. Fiez J. A. Videen T. O. MacLeod A. M. Pardo J. V. Fox P. T.. (1994). Practice-related changes in human brain functional anatomy during nonmotor learning. Cereb. Cortex 4, 8–26. 10.1093/cercor/4.1.8 8180494

[B76] Raven J. C. (1976). Standard Progressive Matrices: Sets A, B, C, D & E. Oxford, UK: Oxford Psychologists Press.

[B77] Rodríguez-Fornells A. Cunillera T. Mestres-Missé A. de Diego-Balaguer R. (2009). Neurophysiological mechanisms involved in language learning in adults. Philos. Trans. R. Soc. Lond. B Biol. Sci. 364, 3711–3735. 10.1098/rstb.2009.0130 19933142 PMC2846313

[B78] Rogalsky C. Rong F. Saberi K. Hickok G. (2011). Functional anatomy of language and music perception: temporal and structural factors investigated using functional magnetic resonance imaging. J. Neurosci. 31, 3843–3852. 10.1523/JNEUROSCI.4515-10.2011 21389239 PMC3066175

[B79] Rugg M. D. (1985). The effects of semantic priming and word repetition on event-related potentials. Psychophysiology 22, 642–647. 10.1111/j.1469-8986.1985.tb01661.x 4089090

[B80] Schellenberg E. G. (2004). Music lessons enhance IQ. Psychol. Sci. 15, 511–514. 10.1111/j.0956-7976.2004.00711.x 15270994

[B81] Schneider P. Scherg M. Dosch H. G. Specht H. J. Gutschalk A. Rupp A. (2002). Morphology of Heschl’s gyrus reflects enhanced activation in the auditory cortex of musicians. Nat. Neurosci. 5, 688–694. 10.1038/nn871 12068300

[B82] Schön D. Magne C. Besson M. (2004). The music of speech: music training facilitates pitch processing in both music and language. Psychophysiology 41, 341–349. 10.1111/1469-8986.00172.x 15102118

[B83] Slevc L. R. (2012). Language and music: sound, structure and meaning. Wiley Interdiscip. Rev. Cogn. Sci. 3, 483–492. 10.1002/wcs.1186 26301531

[B84] Snodgrass J. G. Vanderwart M. (1980). A standardized set of 260 pictures: norms for name agreement, image agreement, familiarity and visual complexity. J. Exp. Psychol. Hum. Learn. Mem. 6, 174–215. 10.1037/0278-7393.6.2.174 7373248

[B85] Strait D. L. Kraus N. Parbery-Clark A. Ashley R. (2010). Musical experience shapes top-down auditory mechanisms: evidence from masking and auditory attention performance. Hear. Res. 261, 22–29. 10.1016/j.heares.2009.12.021 20018234

[B86] Strait D. L. Slater J. O’Connell S. Kraus N. (2015). Music training relates to the development of neural mechanisms of selective auditory attention. Dev. Cogn. Neurosci. 12, 94–104. 10.1016/j.dcn.2015.01.001 25660985 PMC6989776

[B87] Tamminen J. Lambon Ralph M. A. Lewis P. A. (2013). The role of sleep spindles and slow-wave activity in integrating new information in semantic memory. J. Neurosci. 33, 15376–15381. 10.1523/JNEUROSCI.5093-12.2013 24068804 PMC3782619

[B88] Tamminen J. Payne J. D. Stickgold R. Wamsley E. J. Gaskell M. G. (2010). Sleep spindle activity is associated with the integration of new memories and existing knowledge. J. Neurosci. 30, 14356–14360. 10.1523/JNEUROSCI.3028-10.2010 20980591 PMC2989532

[B89] Tervaniemi M. Kruck S. De Baene W. Schröger E. Alter K. Friederici A. D. (2009). Top-down modulation of auditory processing: effects of sound context, musical expertise and attentional focus. Eur. J. Neurosci. 30, 1636–1642. 10.1111/j.1460-9568.2009.06955.x 19821835

[B90] Thompson W. F. Schellenberg E. G. Husain G. (2004). Decoding speech prosody: do music lessons help? Emotion 4, 46–64. 10.1037/1528-3542.4.1.46 15053726

[B91] Torkildsen J. V. K. Friis Hansen H. Svangstu J. M. Smith L. Simonsen H. G. Moen I.. (2009). Brain dynamics of word familiarization in 20-month-olds: effects of productive vocabulary size. Brain Lang. 108, 73–88. 10.1016/j.bandl.2008.09.005 18950850

[B92] Torkildsen J. V. K. Sannerud T. Syversen G. Thormodsen R. Simonsen H. G. Moen I. (2006). Semantic organization of basic-level words in 20-month-olds: an ERP study. J. Neurolinguistics 19, 431–454. 10.1016/j.jneuroling.2006.01.002

[B93] Wagner A. D. Schacter D. L. Rotte M. Koutstaal W. Maril A. Dale A. M.. (1998). Building memories: remembering and forgetting of verbal experiences as predicted by brain activity. Science 281, 1188–1191. 10.1126/science.281.5380.1188 9712582

[B94] Wechsler D. (2003). Wechsler Intelligence Scale for Children (4th Edn.). San Antonio, TX: The Psychological Corporation (WISC-IV).

[B95] Wong P. C. M. Perrachione T. K. (2007). Learning pitch patterns in lexical identification by native English-speaking adults. Appl. Psycholinguist. 28, 565–585. 10.1017/s0142716407070312

[B96] Wong P. C. M. Skoe E. Russo N. M. Dees T. Kraus N. (2007). Musical experience shapes human brainstem encoding of linguistic pitch patterns. Nat. Neurosci. 10, 420–422. 10.1038/nn1872 17351633 PMC4508274

[B97] Zatorre R. J. (2013). Predispositions and plasticity in music and speech learning: neural correlates and implications. Science 342, 585–589. 10.1126/science.1238414 24179219

[B98] Zuk J. Benjamin C. Kenyon A. Gaab N. (2014). Behavioral and neural correlates of executive functioning in musicians and non-musicians. PLoS One 9:e99868. 10.1371/journal.pone.0099868 24937544 PMC4061064

